# Species-Specific Biodegradation of Sporopollenin-Based Microcapsules

**DOI:** 10.1038/s41598-019-46131-w

**Published:** 2019-07-03

**Authors:** Teng-Fei Fan, Michael G. Potroz, Ee-Lin Tan, Mohammed Shahrudin Ibrahim, Eijiro Miyako, Nam-Joon Cho

**Affiliations:** 1 School of Materials Science and Engineering, School of Chemical and Biomedical Engineering, Nanyang Technological University, 50 Nanyang Avenue, 639798 Singapore, Singapore; 2 Department of Materials and Chemistry, Nanomaterials Research Institute (NMRI), National Institute of Advanced Industrial Science and Technology (AIST), Central 5, 1-1-1 Higashi, Tsukuba Ibaraki, 305-8565 Japan

**Keywords:** Biomaterials, Materials chemistry

## Abstract

Sporoderms, the outer layers of plant spores and pollen grains, are some of the most robust biomaterials in nature. In order to evaluate the potential of sporoderms in biomedical applications, we studied the biodegradation in simulated gastrointestinal fluid of sporoderm microcapsules (SDMCs) derived from four different plant species: lycopodium (*Lycopodium clavatum* L.), camellia (*Camellia sinensis* L.), cattail (*Typha angustifolia* L.), and dandelion (*Taraxacum officinale* L.). Dynamic image particle analysis (DIPA) and field-emission scanning electron microscopy (FE-SEM) were used to investigate the morphological characteristics of the capsules, and Fourier-transform infrared (FTIR) spectroscopy was used to evaluate their chemical properties. We found that SDMCs undergo bulk degradation in a species-dependent manner, with camellia SDMCs undergoing the most extensive degradation, and dandelion and lycopodium SDMCs being the most robust.

## Introduction

Sporoderms of pollens are some of the most durable and complex materials in nature owing to their distinct layered structures and chemical properties^1–4^. In a broad sense, the structures and compositions of empty sporoderms, which are sporoderm microcapsules (SDMCs) obtained from different species of plants, are generally similar, composed of an inner (or intine) layer of pectin, cellulose and hemicellulose and an outer (or exine) layer of highly robust sporopollenin^5,6^. In addition to exhibiting favorable species-specific properties such as uniform architectures, narrow size distributions and highly consistent chemical compositions^5–7^, the natural abundance of pollens makes SDMCs a subject of significant interest, especially as natural materials for the microencapsulation of therapeutic agents such as drug compounds^8–17^, proteins^18–23^, peptides^24^, nucleotides^25^, nanoparticles^26,27^, and even living cells^28^.

The potential use of SDMCs as carriers for orally administered drug compounds has been previously investigated using lycopodium sporoderm capsules^24,29,30^, and a growing body of evidence has indicated that intact SDMCs enter the bloodstream and lymph system through the intestinal wall^9,24,30–32^. For example, Atwe *et al*. investigated the effects of orally administered ovalbumin-loaded lycopodium SDMCs in mice, reporting the resultant production of large amounts of anti-ovalbumin IgG in the serum^30^. Their results evidenced the suitability of SDMCs as protein carriers that can effectively shield their payloads from the acidic environment of the gastrointestinal tract. In their study, they also demonstrated the ability of intact SDMCs to cross the intestinal barrier and release their contents into the circulatory system, bringing about systemic effects in the model organism.

While persorption, endocytosis and macrophage engulfment have been proposed as plausible mechanisms for gastrointestinal SDMCs uptake^31^, the release of the contents into the blood plasma has been shown to occur via an enzymatic pathway, leading to the degradation of the SDMCs^8,31^. For example, lycopodium SDMCs loaded with the drug 3,4-diaminopyridine and administered orally to botulism-intoxicated mice were shown to exhibit drug release via a diffusion pathway that is stimulated by intestinal pH conditions^33^.

These findings have great significance for the potential use of SDMCs as drug carriers. However, the biological fate of SDMCs is yet to be fully understood, mainly because the details of the chemical compositions and degradation mechanisms of SDMCs upon oral uptake remain elusive.

Despite being fundamentally similar, sporopollenin from different plant species exhibit significant structural, compositional and property differences owing to their divergent phylogenetic origins. Furthermore, variations in the complex matrix of lipids, carbohydrates and other oxygenated species (*e.g*., ketones, esters, carboxyls and ethers)^2,34–37^ that constitute sporopollenin can lead to differences in the degradation profiles of the resultant SDMCs. Accordingly, addressing this issue would improve our fundamental understanding of sporopollenin chemistry in biological systems, whose knowledge is indispensable for perfecting SDMCs as drug carriers and extending their application to other biomedical fields.

In this study, we investigated the degradation of SDMCs derived from four different plant species—lycopodium (*Lycopodium clavatum* L.) of the lycopodiophyte family, camellia (*Camellia sinensis* L.) and dandelion (*Taraxacum officinale* L.) of the dicots family, and cattail (*Typha angustifolia* L.) of the monocots family^38^—using simulated gastric fluid (SGF) and simulated intestinal fluid (SIF) as a model for the conditions of the human digestive system. These four species were selected because they are commercially harvested and thus easily obtained. Dynamic image particle analysis (DIPA) and field-emission scanning electron microscopy (FE-SEM) were used to investigate the morphologies of the SDMCs, and Fourier-transform infrared (FTIR) spectroscopy was used to investigate the chemical bonds in the SDMCs. We believe that the findings reported herein are invaluable for understanding the degradation mechanism of different SDMCs in biological systems, as well as constituting a scientific basis for the future biomedical application of SDMCs.

## Materials and Methods

### Materials

Natural *Lycopodium clavatum* spores (S-type) were purchased from Sigma-Aldrich Co., LLC (St. Louis, MO, USA). *Camellia sinensis* (camellia) bee pollen was purchased from Xi’an Yuenun Biological Technology Co., Ltd. (Xi’an, China). Natural *Typha angustifolia* (cattail) pollen grains were sieved from cattail flowers purchased from Wong Yiu Nam Medical Hall Pte. Ltd. (Singapore). Defatted *Taraxacum officinale* (dandelion) pollen grains were purchased from Greer Labs (Lenoir, NC, USA). All solvents and reagents were obtained from Sigma-Aldrich Co., LLC, nylon mesh was purchased from ELKO Filtering Co., LLC (Miami, FL, USA) and 50 µm Duke Standards polystyrene microspheres were purchased from Thermo Fisher Scientific Pte. Ltd. (Waltham, MA, USA).

### Preparation of SDMCs

The preparation method of SDMCs from camellia, cattail and dandelion was the same as that previously reported for lycopodium with slight modifications^18^.

#### Lycopodium clavatum (Lycopodium)

Briefly, natural *L. clavatum* spores (100 g) were defatted by refluxing in acetone (500 mL) at 50 °C for 6 h under stirring (220 rpm). The defatted spores were then collected by vacuum filtration and air-dried for 12 h. The dried samples were then refluxed (70 °C) in an aqueous 6% (w/v) potassium hydroxide solution (500 mL) under stirring for 6 h. The samples were collected by vacuum filtration and washed with Milli-Q water (Merck Millipore, MA, Burlington, USA) (2 × 500 mL) before resuming alkaline lysis for another 6 h using a 6% (w/v) potassium hydroxide solution (500 mL). After 12 h of alkaline lysis, the SDMCs were collected by centrifugation at 4,500 rpm and washed with hot Milli-Q water (5 × 500 mL, 50 °C). After each wash, the suspension was collected by vacuum filtration. The SDMCs were then washed with hot ethanol (2 × 500 mL, 50 °C) and dried overnight under fume hood. The resultant SDMCs were subjected to acidolysis by suspension in 85% (v/v) phosphoric acid (500 mL) and stirring under gentle reflux at 70 °C for 5 h. After acidolysis, the SDMCs were collected and washed sequentially in hot water (5 × 800 mL, 50 °C), hot acetone (600 mL, 50 °C), hot 2 M hydrochloric acid (600 mL, 50 °C), hot 2 M sodium hydroxide (600 mL, 50 °C), hot water (5 × 800 mL, 50 °C), hot acetone (600 mL, 50 °C) and then hot ethanol (600 mL, 50 °C). The resulting SDMCs were finally collected by vacuum filtration. The washed SDMCs were transferred to a clean glass dish and air-dried for 12 h. Drying was completed in a vacuum oven (Memmert, Schwabach, Germany) at 60 °C for 8 h, and the dried SDMCs were then stored in a dry cabinet.

#### Camellia sinensis (Camellia)

Similarly, a defatting process was performed by refluxing camellia bee pollen (250 g) in hot acetone (500 mL, 50 °C) with magnetic stirring (220 rpm, 3 h). The acetone was removed by vacuum filtration, and 1 L of warm water was added to the pollen under stirring. The resulting mixture was passed through a 150 µm nylon mesh (ELKO Filtering Co., LLC) to remove any contaminants. Water was removed from the suspension via vacuum filtration. This process was then repeated for another wash cycle.

The purified pollen was refluxed again in acetone (500 mL, 50 °C), isolated by vacuum filtration, transferred to a glass dish, and air-dried under fume hood. The dry pollen powder (20 g) was resuspended in diethyl ether (250 mL) under stirring (300 rpm, 2 h) at room temperature. The process was repeated for another wash cycle using fresh diethyl ether. For the final wash, the pollen was added to diethyl ether (500 mL) and stirred (300 rpm) at room temperature overnight. The defatted pollen was isolated by vacuum filtration and left to dry under fume hood.

The defatted pollen (6 g) was refluxed in 85% (v/v) phosphoric acid (60 mL) at 70 °C for 5 h (220 rpm). The resulting SDMCs were filtered and washed sequentially with 50 mL portions of water (five times), acetone (twice), 2 M hydrochloric acid (once), 2 M sodium hydroxide (once), water (five times), acetone (once), ethanol (twice) and water (once). The samples were dried under fume hood overnight and then in a vacuum oven (60 °C, 4 h). The dried SDMCs were stored in a dry cabinet at room temperature until further characterization.

#### Typha angustifolia (Cattail)

Natural cattail pollen grains (10 g) were defatted by refluxing in acetone (100 mL) at 45 °C with magnetic stirring (200 rpm, 30 min). The pollen grains were then obtained by vacuum filtration and washed with acetone (50 mL). The defatted pollen grains were then dried under fume hood at room temperature for 12 h.

To isolate the SDMCs via acidolysis, the defatted pollen grains (2 g) were placed in a poly(tetrafluoroethylene) round-bottom flask containing 85% (v/v) phosphoric acid (15 mL) and refluxed at 70 °C (water bath) for 2.5 h under gentle magnetic stirring (180–200 rpm). After 2.5 h, the flask was removed from reflux and its contents were allowed to cool down to room temperature. The suspension was then diluted with deionized water (150 mL) and vacuum-filtered. The SDMCs were collected in a clean 250 mL beaker and washed with 150 mL of warm water. The warm water wash was repeated five times with vacuum filtration until the pH of the washings reached approximately 6 or 7. The resulting SDMCs were collected in a clean 250 mL beaker, and washing steps were conducted by bathing the capsules sequentially in 100 mL portions of hot acetone (twice), hot 2 M hydrochloric acid (once), hot 2 M sodium hydroxide (once), hot water (five times), hot acetone (once), hot ethanol (twice) and finally hot water (three times). During each wash, the SDMCs were stirred in a beaker to produce a homogenous mixture and to prevent the capsules from aggregating. The solvent was then removed by vacuum filtration. The final washed capsules were then transferred to a clean glass dish and air-dried at room temperature for 12 h.

#### Taraxacum officinale (Dandelion)

Defatted dandelion pollens (2 g) were mixed with 85% (v/v) phosphoric acid (15 mL) in a 50 mL single-neck flask fitted with a glass condenser and refluxed at 70 °C for 5 h under magnetic stirring (220 rpm). After acidolysis, the SDMCs were collected by vacuum filtration and washed sequentially with 100 mL portions of hot water (five times, 50 °C), hot acetone (twice, 50 °C), hot 2 M hydrochloric acid (once, 50 °C), hot 2 M sodium hydroxide (once, 50 °C), hot water (five times, 50 °C), hot acetone (once, 50 °C), hot ethanol (twice, 50 °C), and hot water (once, 50 °C). The final product was collected by vacuum filtration, and the washed SDMCs were then transferred to a clean glass dishand air-dried under fume hood overnight. The drying process was continued in a vacuum oven at 60 °C under 1 mbar vacuum conditions for 4 h. Finally, the dried SDMCs were stored in a dry cabinet at room temperature for further characterization.

### Preparation of simulated human gastrointestinal fluids

The conditions of physiological digestion areas described in the literature^39^. For SGFs containing an enzyme, 0.2 wt.% NaCl and 0.32 wt.% pepsin were dissolved in deionized water, and the pH was adjusted to 2.0 by adding 0.2 M HCl to the solution. For SIFs containing an enzyme, 0.68 wt.% monobasic potassium phosphate and 1 wt.% pancreatin were dissolved in deionized water, and the pH was adjusted to 7.1 using 0.2 M NaOH and, in the case of overshoot,0.2 M HCl.

### Degradation treatment of SDMCs

The SDMCs were divided into three groups that were subjected to different degradation conditions: SGF treatment, SIF treatment and a control group in which water was used as the incubation medium. For SGF and SIF treatment, the SDMCs were segmented into two 40 mg batches, each of which was loaded into a 2 mL Eppendorf tube containing 0.6 mL of SGF or SIF, mixed thoroughly by vortexing for 1 min, and incubated at 37 °C in an orbital shaker incubator (LM-450D, Yihder Technology Co., Ltd., New Taipei City, Taiwan) with circular shaking at 220 rpm. For the control group, the SDMCs (40 mg) were incubated in 0.6 mL of deionized water for 24 h under the same incubation conditions. These three batches of SDMCs were then sampled at set durations (e.g., 1 h, 24 h), isolated by centrifugation at 7,000 rpm for 15 min, washed with SGF or SIF (1 mL) for five times and deionized water (1 mL) for five times and lyophilized.

### DIPA

DIPA was performed using a FlowCAM VS (Fluid Imaging Technologies, Scarborough, ME, USA) equipped with a 200 μm flow cell (FC-200) and a 20x magnification lens (Olympus, Tokyo, Japan). After 0.5 mL of prerun, untreated, SGF-treated and SIF-treated SDMCs samples were primed manually into the flow cell at 2 mg mL^−1^. Imaging was performed at a flow rate of 0.1 mL min^−1^ and a camera rate of 14 frames s^−1^. At least 10,000 particles were counted for each measurement, and three separate measurements were performed per sample. One thousand well-focused SDMCs were selected by edge gradient ordering and manual processing for the representative images.

### FTIR spectroscopy

FTIR measurement was performed with a PerkinElmer Spectrum (PerkinElmer, Seer Green, Buckinghamshire, UK) equipped with a diamond cell attenuated total reflection accessory. Reflectance infrared (IR) spectra were collected in the midinfrared region of 4,000–650 cm^−1^with 16 scans per measurement and 6 replicate measurements per sample.

Background spectra were collected before sample analysis and automatically subtracted from each measurement. Baseline correction was carried out using Spectrum 10 software (PerkinElmer). After baseline correction, each spectrum was standardized as previously reported. Briefly, $(x-\bar{x})/\sigma$ values were used, where *x* refers to the absorbance value, $\bar{x}$ refers to the spectrum arithmetic mean, and σ refers to the spectrum standard deviation. Peak heights were measured by taking the maximum value within a given range (Table 1). The peak ratios among the various peaks were calculated to remove the potential effect of differences in sample thickness on the absolute absorbance values. Principal component analysis (PCA) was performed using Origin 2018 (OriginLab, North Hampton, MA, USA).

**Table 1:** Assignments of absorbance peaks in the FTIR spectra of three species of SDMCs.

Wavenumber (cm^−1^)	Assignment	Interpretation
3,370–3,384	νO–H	Hydroxyl
2,925	νCH_2_	Aliphatic (lipid, sporopollenin)
1,745	νC=O	Lipid
1,673–1,700	νC=O	Lipid, sporopollenin
1,516	νC=C	Aromatic compounds
1,104–1,060	νC–O–C	Carbohydrate

### FE-SEM

Untreated and SGF/SIF-treated SDMCs samples were frozen and lyophilized overnight in a freeze dryer (Labconco, Kansas City, MO, USA) under 0.008 mbar vacuum. A small number of samples were immobilized on a sample holder with carbon tape and sputter-coated with gold to a thickness of 20 nm (20 mA, 80 s) using a JFC-1600 Auto Fine Coater (JEOL, Tokyo, Japan) to reduce charging effects during SEM imaging. FE-SEM images were taken with a JSM-7600F Schottky microscope (JEOL) at an acceleration voltage of 5.00 kV. Visual inspection of more than 50 randomly chosen SDMCs particles from all the untreated and SGF/SIF-treated SDMCs samples was performed at different magnifications (500x, 2,500x and 15,000x) to assess any morphological changes in the samples.

## Results and Discussion

**Figure 1:**
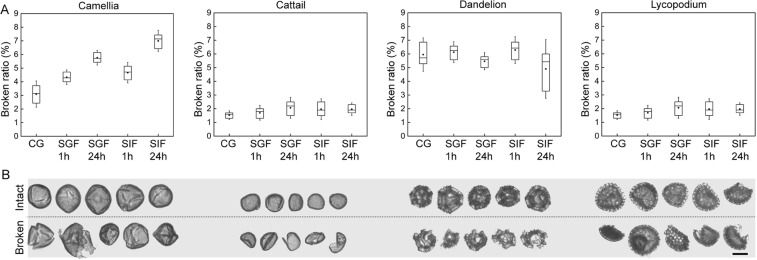
DIPA analysis of the four SDMCs after incubation in simulated gastrointestinal fluids. (**A**) Boxplot representations of the broken particle population for the four SDMCs. Dots indicate the median and the whiskers indicate the highest and lowest points within 1.5 standard deviations. The broken particle population of untreated samples served as a control for each SDMCs species. (**B**) Representative DIPA images of intact and broken SDMCs.

In order to investigate the morphological nature of the SDMCs, we used DIPA to characterize the four SDMCs species (Fig. 1). The SDMCs diameters and aspect ratios are summarized in Table S1. SDMCs particles that showed signs of cracks, noninherent large holes, or partial wall sectors were deemed broken. In comparison to the untreated group, the SDMCs incubated in SGF and SIF exhibited only slight differences in diameter, aspect and circularity. However, broken SDMCs can still be counted by visual inspection of the optical micrographs of each individual particle. As demonstrated in Fig. 1B, representative examples of intact and broken particles were assigned to each group. Cracks, large holes or partial wall sectors were all termed ‘broken’, and the ‘broken’ ratio was calculated for each group. Most of the broken camellia and cattail SDMCs remained as single particles, whereas the dandelion and lycopodium SDMCs were observed to break into several pieces (Fig. 1B).

**Figure 2:**
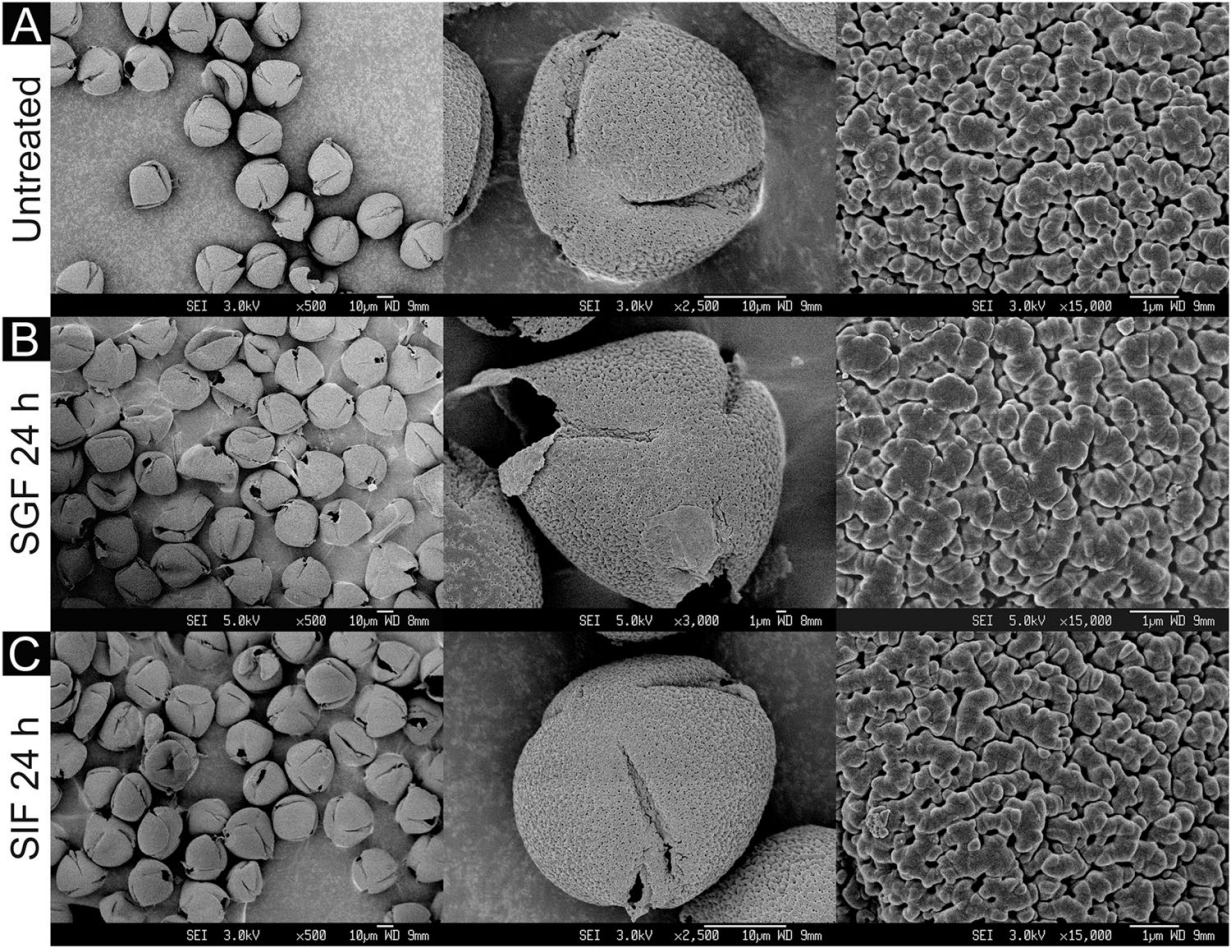
SEM images of camellia SDMCs before and after degradation treatment with simulated gastrointestinal fluids (SGF and SIF). Surface morphology of (**A**) untreated camellia SDMCs and those treated for 24 h with (**B**) SGF or (**C**) SIF, at different magnifications.

**Figure 3:**
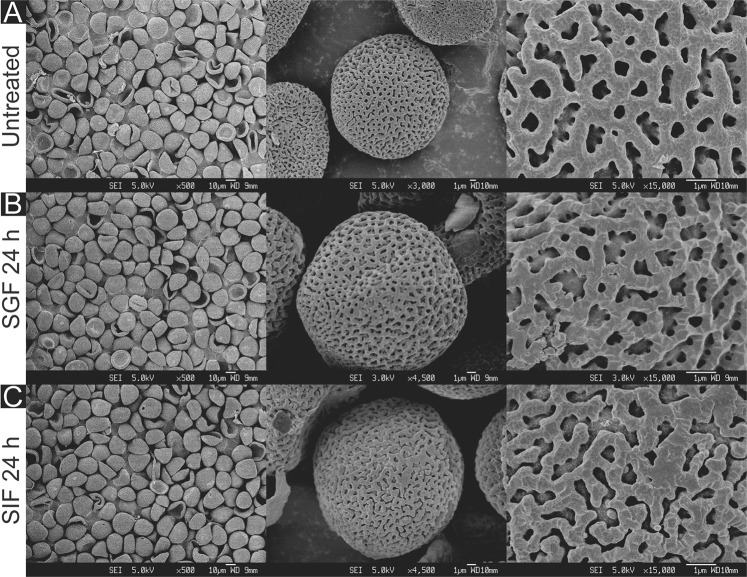
SEM images of cattail SDMCs before and after degradation treatment with simulated gastrointestinal fluids (SGF and SIF). Surface morphology of (**A**) untreated cattail SDMCs and those treated for 24 h with (**B**) SGF or (**C**) SIF, at different magnifications.

**Figure 4:**
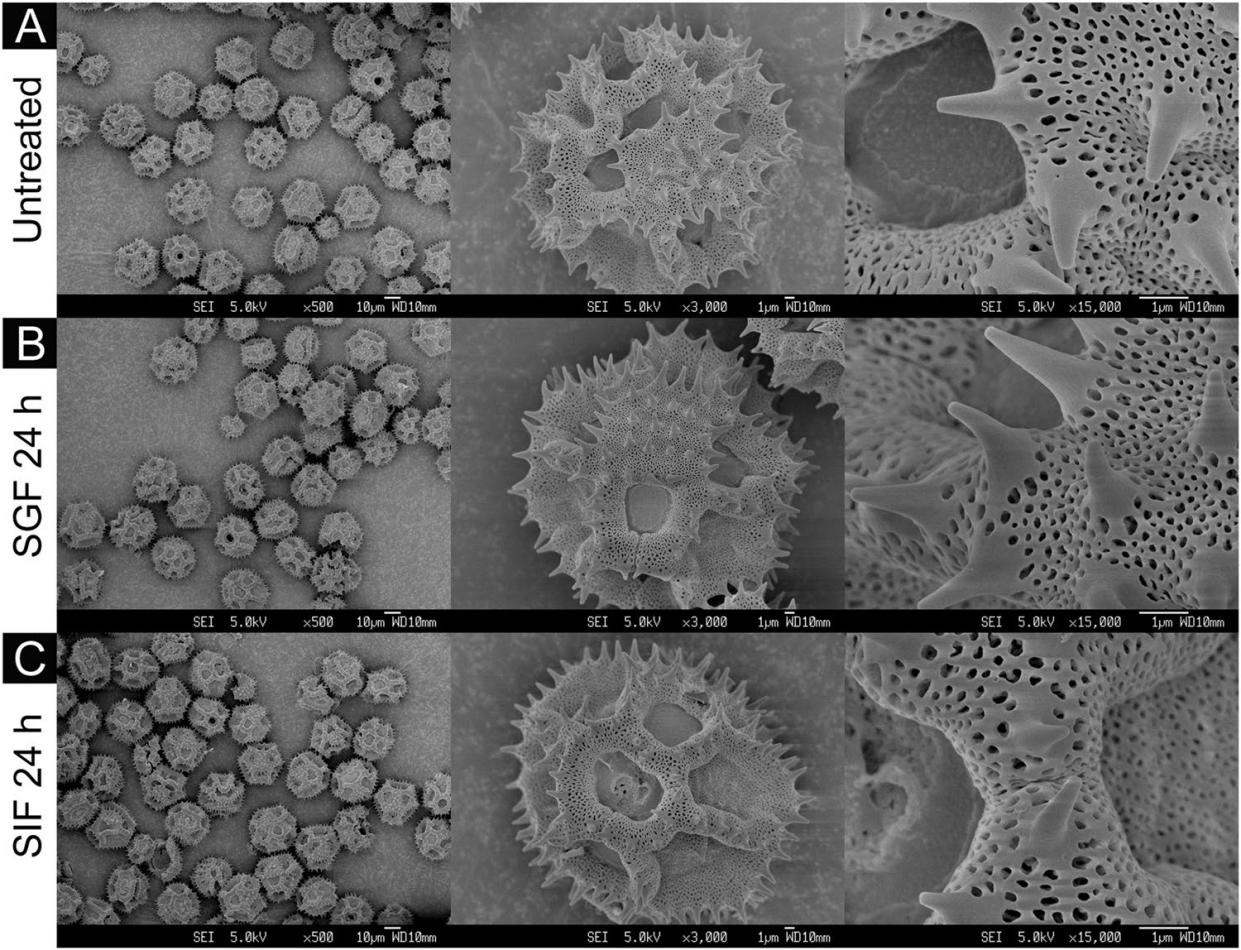
SEM images of dandelion SDMCs before and after degradation treatment with simulated gastrointestinal fluids (SGF and SIF). Surface morphology of (**A**) untreated dandelion SDMCs and those treated for 24 h with (**B**) SGF or (**C**) SIF, at different magnifications.

**Figure 5:**
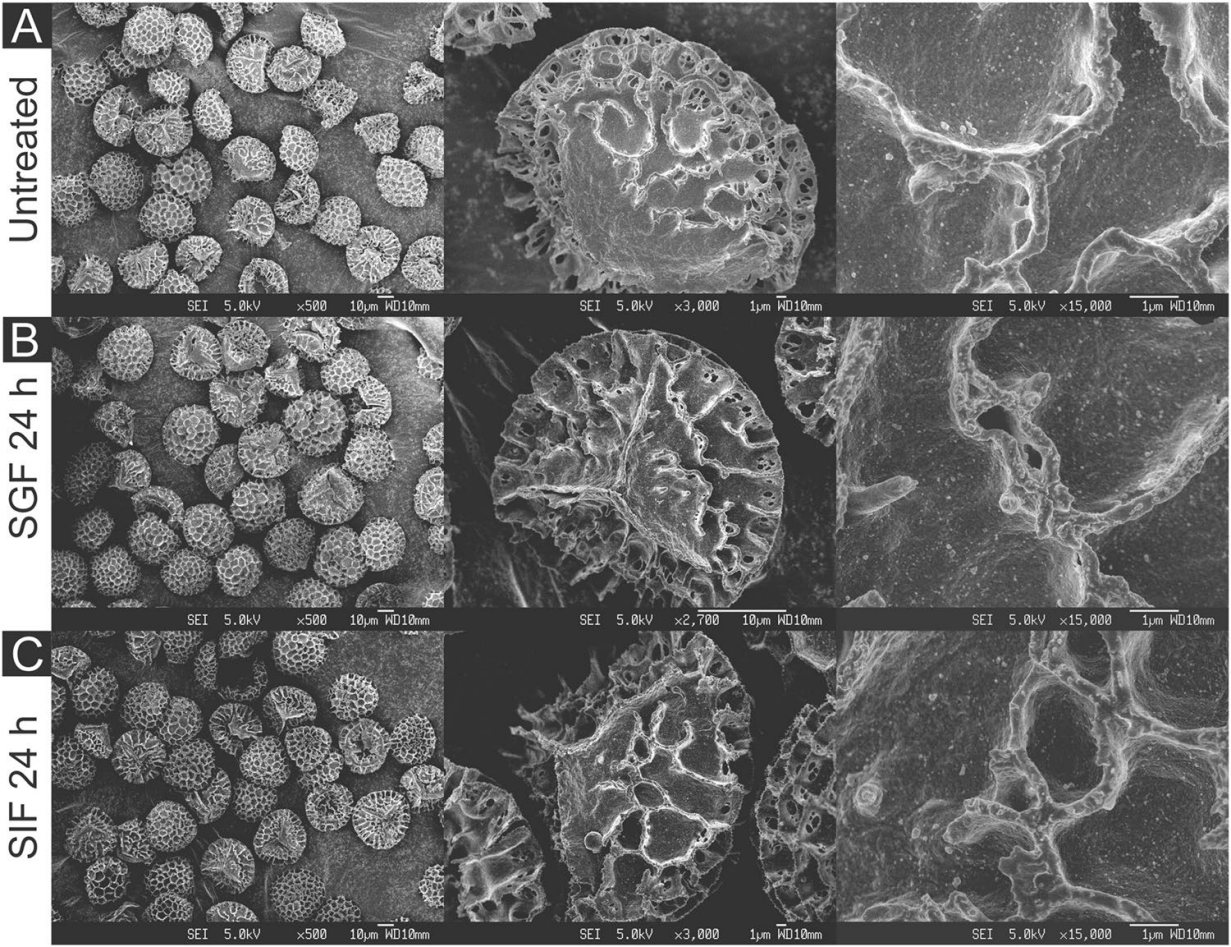
SEM images of lycopodium SDMCs before and after degradation treatment with simulated gastrointestinal fluids (SGF and SIF). Surface morphology of (**A**) untreated lycopodium SDMCs and those treated for 24 h with (**B**) SGF or (**C**) SIF, at different magnifications.

The impressive morphological variation of pollen grains between various plant species was immediately evident upon the analysis of the physical characteristics of the four SDMCs. Of particular interest were the unique geometries of the apertures, which are the weakest site of the pollen outer wall^40^. Camellia SDMCs are triaperturate, with three cleavage planes (Fig. 2B). Cattail SDMCs are monopartite (Fig. 3B), dandelion SDMCs possess three endoapertures that are surrounded by ridges (Fig. 4B), and lycopodium SDMCs are trilete (Fig. 5B). Most breakages on the camellia SDMCs were observed along the aperture stripe, which suggests that the walls of the camellia SDMCs might be weakened and torn away during the incubation and washing processes, whereas only small holes were formed on the surface of the cattail SDMCs without bulk erosion. Given that the number, arrangement and shape of the apertures greatly influence the mechanical properties of the pollen grains^40–42^, it seems highly likely that the relative fragility of the camellia SDMCs can be attributed to its distinct aperture geometry.

The surface morphologies of the four SDMCs were examined for signs of surface degradation after SGF or SIF treatment. Camellia SDMCs exhibited a nanoparticle-assembled porous surface structure (Fig. 2C), cattail SDMCs exhibited a honeycombed surface with large irregular passages (Fig. 3C), dandelion SDMCs exhibited porous echinate ridges (Fig. 4C) and lycopodium SDMCs exhibited web-like microridges and a tripartite structure (Fig. 5C). We observed that the surface morphologies of the SDMCs remain largely unchanged after incubation for 24 h in SGF or SIF (Figs 1–4). This suggests that surface erosion does not play a major role in SDMCs degradation, concurring with previously reported findings on lycopodium SDMCs degradation in human blood plasma^8,24^.

**Figure 6:**
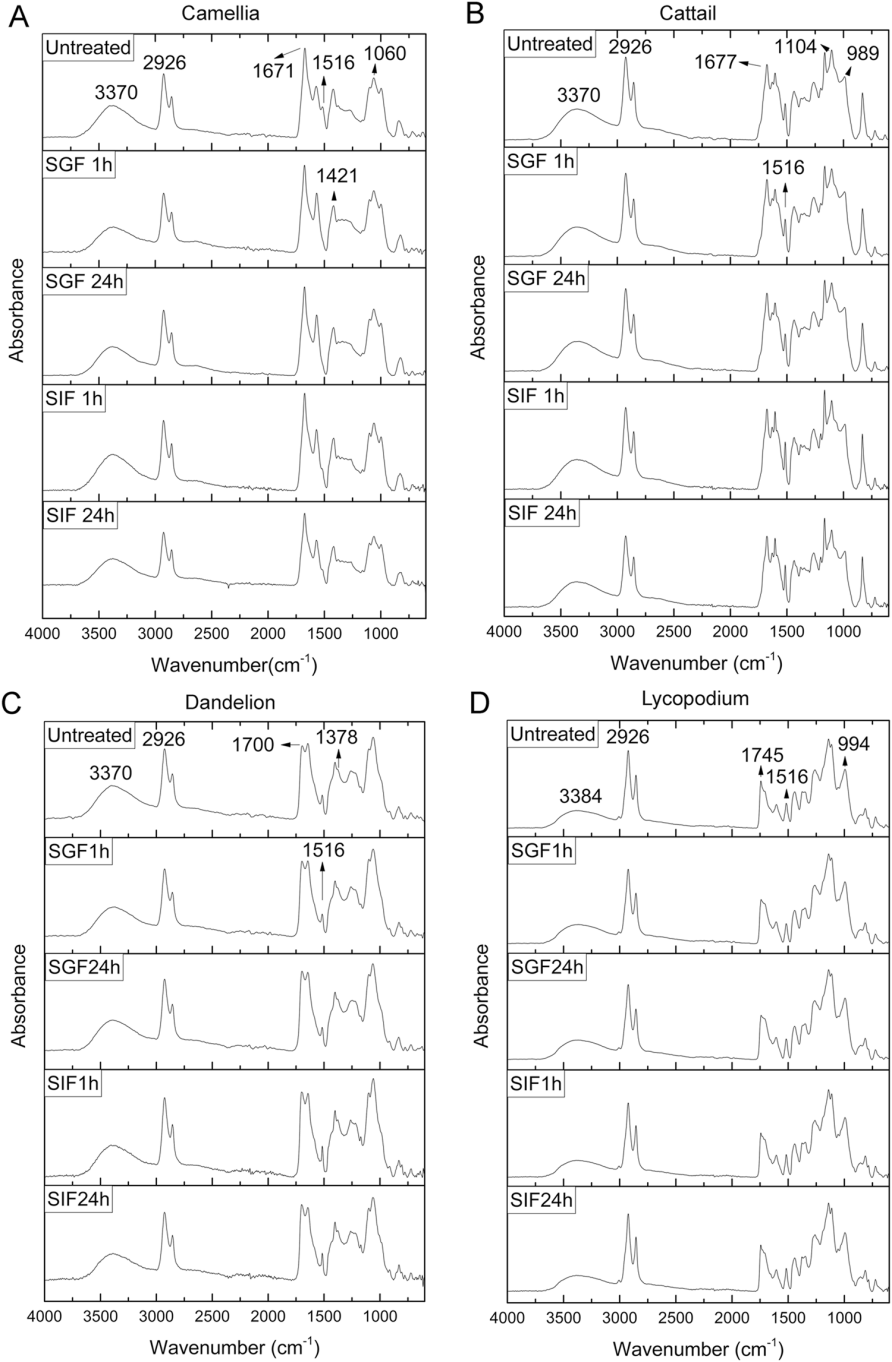
FTIR spectra of SDMCs before and after incubation in SGF/SIF. The spectra presented are the means of six replicates. (**A**) Camellia, (**B**) cattail, (**C**) dandelion, and (**D**) lycopodium.

We performed a comprehensive analysis of the FTIR spectra collected for the untreated and incubated SDMCs in order to identify any chemical changes and possible pathways of degradation (Fig. 6). To remove the effects of the sample thickness on the peak height, all the FTIR spectra were standardized to zero mean and unit variance (*z*-scores)^35^. The resultant functional group peaks for the SDMCs were assigned according to the literature^35,37,43–49^ and are summarized in Table 1. Briefly, a common peak at 1,516 cm^−1^ is observed for all four species of SDMCs, which is an attribution of UV sensitive aromatic rings in spropollenin^35^. Several peaks attributed to functionalized carbohydrates (e.g., C–O–C, C–OH) were observed between 1,200 and 900 cm^−1^, with the peak shapes varying between the different species. A strong peak at 1,745 cm^−1^, representing lipids, is observed only for the lycopodium SDMCs. Overall, as expected, the spectral differences between the four species of SDMCs, especially between 800 and 1,800 cm^−1^, were attributed to their different compositions.

**Figure 7:**
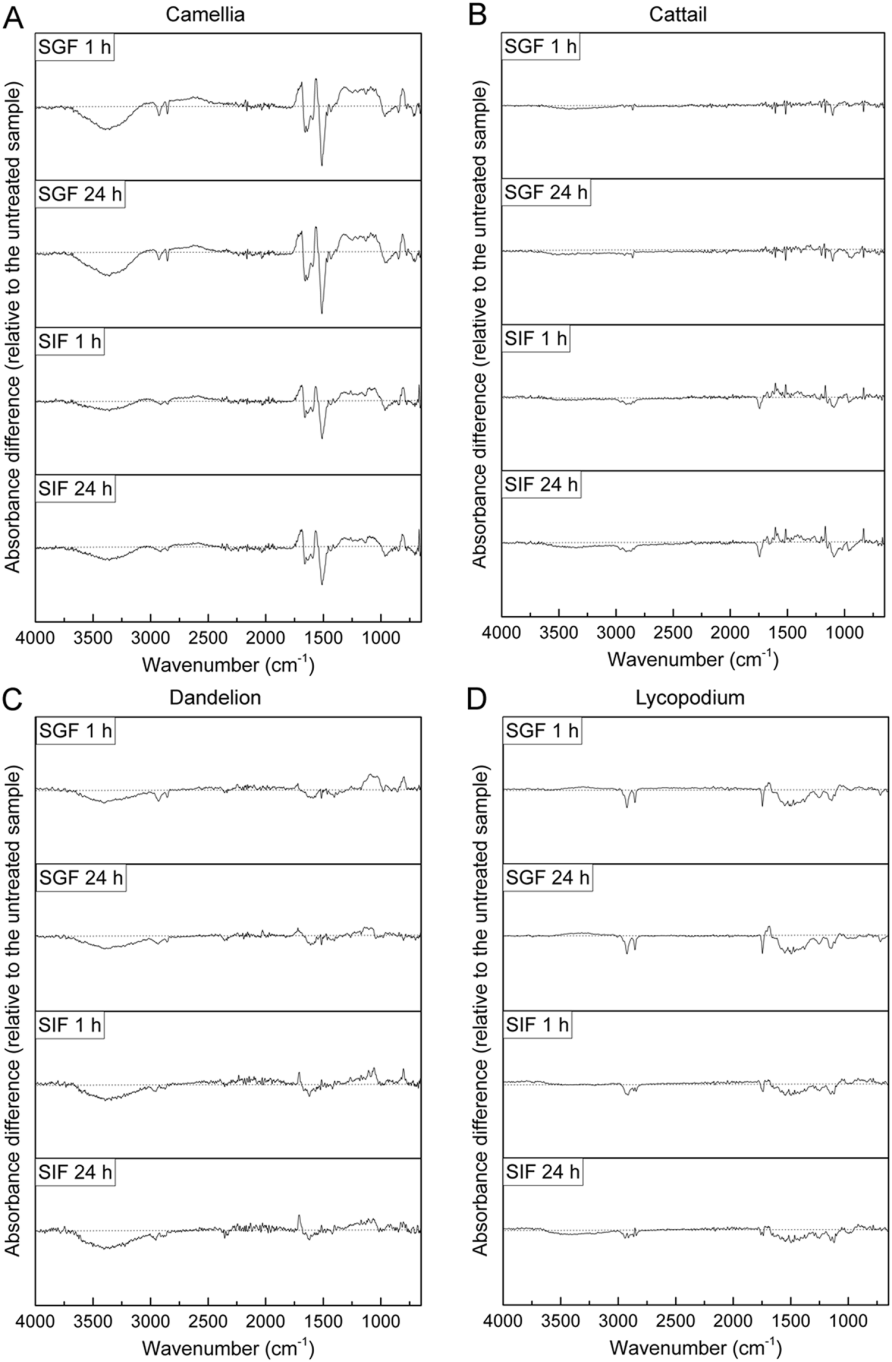
FTIR absorbance difference spectra of the four species of SDMCs after degradation treatment. Each plot was produced with the treated sample mean spectrum minus the mean untreated spectrum. The gray dashed lines indicate the0 value. (**A**) Camellia, (**B**) cattail, (**C**) dandelion, and (**D**) lycopodium.

After incubation treatment, no significant changes in peak shape or number were observed, except for the 1,516 cm^−1^ peak in camellia SDMCs, which was absent after 1 h of incubation in SGF. The absorbance difference spectra, as presented in Fig. 7, further highlight the more noticeable change in the camellia SDMCs relative to the other SDMCs. Here, the FTIR absorbance difference spectrum of the camellia SDMCs was the most changed upon degradation treatment, with its hydroxyl, aliphatic and aromatic group peaks exhibiting significant decreases in peak intensity, whereas the carboxyl group peaks and the peaks in the 1,400–1,000 cm^−1^ range were seen to increase, likely due to the formation of new bonds, such as C–O bond. Similar results regarding the formation of new C–O bonds during the acetolysis of sporopollenin have been reported^37^. Cattail SDMCs appear to be the most stable and show the least fluctuation in their FTIR absorbance difference spectra. The hydroxyl groups in the lycopodium SDMCs were relatively stable in both SGF and SIF, whereas the other peaks presented a decreasing trend. These results demonstrate that the chemical signatures of SDMCs are altered to various degrees during incubation in gastrointestinal fluids, most likely owing to their species-dependent chemical composition.

**Figure 8:**
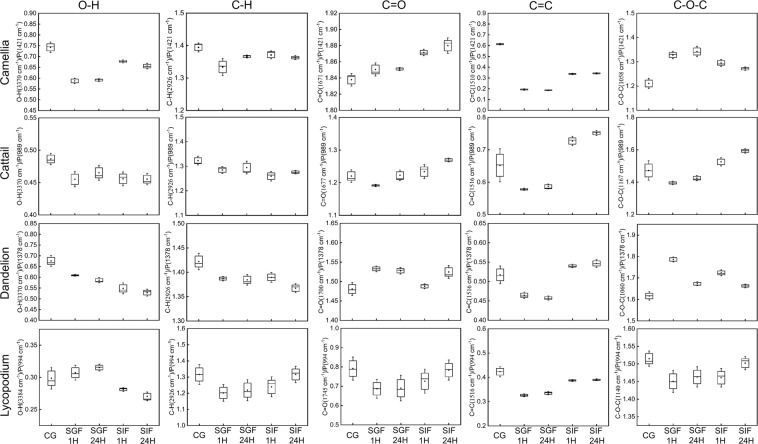
Boxplot representations of peak height ratios for the different functional groups in the four species of SDMCs before and after incubation. Internal standard peaks were set as 1,421 cm^−1^ for camellia, 989 cm^−1^ for cattail, 1,378 cm^−1^ for dandelion, and 994 cm^−1^ for lycopodium. The dots indicate the median and whiskers indicate the highest and lowest points within 1.5 standard deviations.

**Figure 9:**
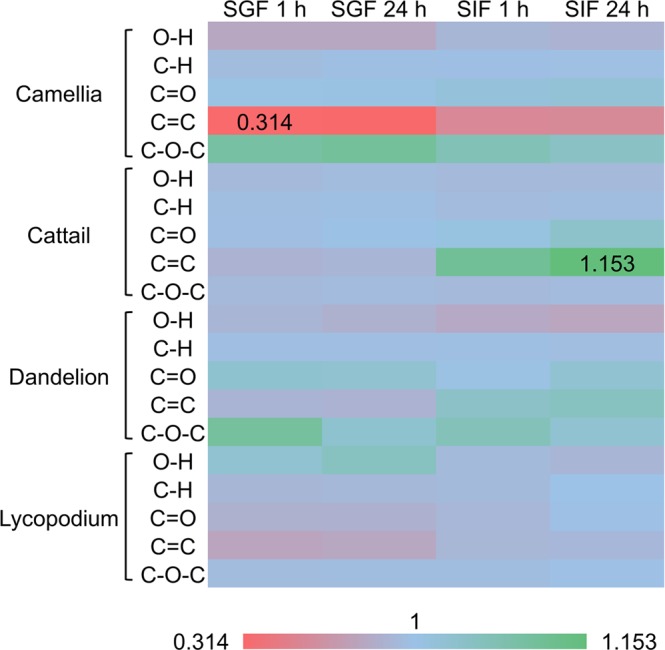
Comparison of the normalized peak height ratios for different functional groups in the four species of SDMCs after different treatments. The color of the bar changes from red to blue, indicating that the peak ratio values increase from the minimum (0.314) to the maximum (1.153). For each SDMCs species, the peak ratio of each corresponding functional group was set as 1 (not shown). The peak ratio was the mean value of six replicates.

Semiquantitative analysis of peak heights was further performed by calculating the peak height ratios for different SDMCs functional groups (Fig. 8). Different internal standard peaks were used for each species, and the peak that showed the least variation in peak height and wavenumber was chosen. Typically, the 1,421 cm^−1^ peak was set as the internal standard for camellia, 989 cm^−1^ for cattail, 1,378 cm^−1^ for dandelion and 994 cm^−1^ for lycopodium. The peak heights for C = O, C = O, C–H, C–O–C and O–H groups were then standardized relative to the corresponding internal standard. The peak ratios were normalized relative to the untreated SDMCs, and a heat map was generated to better compare the chemical stability of all four SDMCs (Fig. 9).

In order to further normalize and visualize the changes in peak height ratios, a heat map was constructed to represent the data (see Fig. 9). For the treated samples, the peak height ratios of the abovementioned functional groups were normalized to that of the untreated samples. C = C groups were seen to be unstable in all four SDMCs when incubated in SGF for 1 h. The greatest change was observed for camellia, which presented a decrease of ≈ 0.3 in the peak height ratio. A similar phenomenon has been reported for lycopodium sporopollenin, in which UV-B-absorbing compounds that contain double bonds were removed by oxidation^49^. This suggests that enzymatic lysis in gastric fluids could play a similar role for the plant SDMCs. However, upon treatment in SIF, the changes in the peak height ratios are more complex. While cattail and dandelion presented an increase in the C = C peak height ratio, a decrease was observed for camellia SDMCs, and no change was observed for lycopodium SDMCs. These results demonstrate that even though the main sporopollenin component of the SDMCs is common to a range of plant species, the diversity in the quantity and chemistry of its functional groups significantly affect its biodegradability.

**Figure 10:**
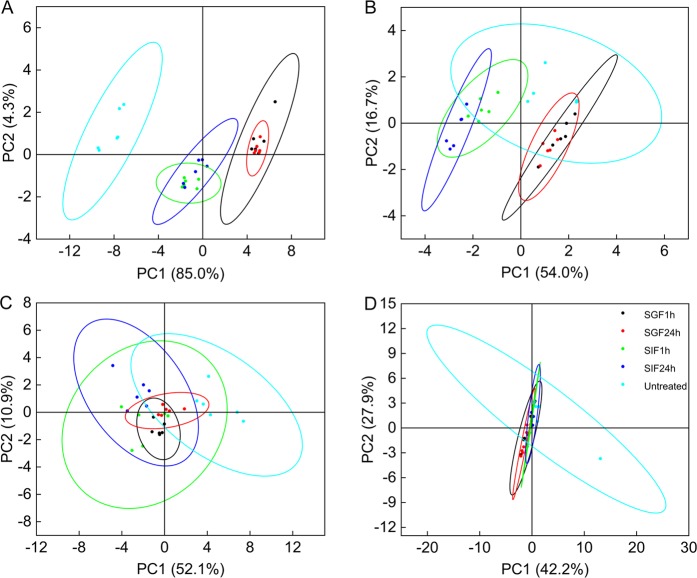
PCA of SDMCs before and after incubation in gastrointestinal fluids. (**A**) Camellia, (**B**) cattail, (**C**) dandelion, and (**D**) lycopodium. The cyan dots denote untreated samples, the blue dots denote samples treated with SIF for 1 h, the green dots denote samples treated with SIF for 24 h, the red dots denote samples treated with SGF for 1 h, and the black dots denote samples treated with SGF for 24 h. The ellipses represent 95% confidence intervals for each group.

Finally, PCA of the FTIR spectra was performed to observe data clustering for the untreated, SGF-treated and SIF-treated samples (Fig. 10). PCA is an ordinary multivariate analysis technique for data dimensionality reduction, thereby identifying the differences and similarities among the samples and the variables that constitute the modeled data^50,51^. The aim of PCA is to obtain a small set of principal components (PCs) that explain the most variable parameters of the data sets. This method has proved useful in the interpretation of FTIR spectra, which show band diversity and complication depending on the source of the sample^52,53^. In this work, standardized FTIR data sets, which consist of the wavenumber and the corresponding absorbance, were used to perform PCA. The first PC describes as much of the variability (variation of absorbances) in the data as possible, the second PC, orthogonal to the first, accounts for as much of the remaining variability as possible, and so forth^49,52,53^. We observed that the first two components account for more than 60% of the total variance for each species.

The results of the PCA are presented in Fig. 10. Compounding our earlier results, only camellia shows a clear difference in IR spectra between the untreated control and the SGF- or SIF-treated SDMCs. Here, the first component (PC1) accounts for 85% of the variance in the FTIR database, thereby separating the untreated control from the SGF and SIF groups, mostly owing to a decrease in the 3,370 cm^−1^ and 1,516 cm^−1^ peaks (Fig. 7). The second component (PC2)  accounted for only 4.3% of the variance, relating to fluctuations in the 3,370 cm^−1 ^band and some weak peaks between 600 and 800 cm^−1^.

For cattails, although the SGF/SIF groups cannot be separated from the untreated control, SGF and SIF were separated at the 5% level of significance. The sums of variance, as presented by PC1 and PC2, are 54% and 16.7% (Fig. 10). Here, a clear separation is seen between SGF and SIF degradation (Fig. 10). For dandelion and lycopodium, all three groups overlap, indicating that the chemical changes here were not significant enough to be separated out by PCA. Thus, it may be inferred that dandelion and lycopodium SDMCs  were stable during incubation with simulated gastrointestinal fluids. Finally, it was observed that prolonged incubation times in both SGF and SIF have no further significant effects on the SDMCs chemical signatures, indicating that most of the chemical changes/degradation occured within the first hour.

Thus, combining the results of PCA and peak height ratio calculations, we conclude that the stability and quantity of functional groups are dependent on differences in the composition and properties of the SDMCs obtained from different plant species. Intriguingly, although the FTIR and PCA analysis showed that chemical changes occur within the first hour of incubation (e.g., camellia SDMCs), the morphology of these SDMCs  remained consistent. As surface erosion leads to the loss of surface material, while bulk erosion results in rapid internal degradation^54^, we propose that bulk erosion is the main mechanism for the degradation of SDMCs in SGF and SIF^54,55^.

## Conclusions

In this study, the degradation of SDMCs obtained from four different species of plants (camellia, cattail, dandelion and lycopodium) in simulated human gastrointestinal fluids was studied. Physical characterization of the SDMCs was performed using FE-SEM and DIPA, and their chemical compositions were studied using FTIR spectroscopy, and the resulting data were analyzed using PCA. The four species of SDMCs showed various degrees of chemical degradation; camellia SDMCs were observed to undergo the most significant degradation upon incubation in SGF and SIF, whereas dandelion and lycopodium SDMCs appeared to remain stable. The analysis of our results indicated that bulk erosion is the main mechanism for the degradation of SDMCs in SGF and SIF. Overall, we have demonstrated that the degradation of different SDMCs occurs in a species-dependent manner in response to physiological conditions. We believe that these findings provide insights and understanding that will aid future biological applications of SDMCs.

## Supplementary information

Table S1
