# Supplementary material for: Species-Specific Biodegradation of Sporopollenin-Based Microcapsules
Source: Sci Rep. 2019 Jul 3;9:9626. doi: 10.1038/s41598-019-46131-w (PMC6610089; doi:10.1038/s41598-019-46131-w)
Supplement: Supplementary file 1 — Table S1 [file 41598_2019_46131_MOESM1_ESM.docx]

**Supplementary Information**

**Species-Specific Biodegradation of Sporopollenin-Based Microcapsules**

Teng-Fei Fan,^1^ Michael G. Potroz,^1^ Ee-Lin Tan,^1^ Mohammed Shahrudin Ibrahim,^1^ Eijiro Miyako^2^ and Nam-Joon Cho^1^*

^1^School of Materials Science and Engineering, School of Chemical and Biomolecular Engineering, Nanyang Technological University, 50 Nanyang Avenue 639798, Singapore

^2^Department of Materials and Chemistry, Nanomaterials Research Institute (NMRI), National Institute of Advanced Industrial Science and Technology (AIST), Central 5, 1-1-1 Higashi, Tsukuba, Ibaraki 305-8565, Japan

E-mail: [njcho@ntu.edu.sg](mailto:njcho@ntu.edu.sg)

**Table S1:** Morphological data for the four SDMCs before and after degradation treatment.

| Species | Treatment | Diameter (µm) | Aspect ratio | Circularity |
| --- | --- | --- | --- | --- |
| Camellia | Control group | 31.85 ± 4.19 | 0.87 ± 0.10 | 0.97 ± 0.09 |
|  | SGF, 24 h | 33.42 ± 5.14 | 0.86 ± 0.11 | 0.96 ± 0.10 |
|  | SIF, 24 h | 31.42 ± 4.02 | 0.85 ± 0.10 | 0.96 ± 0.08 |
| Cattail | Control group | 19.61 ± 1.91 | 0.80 ± 0.11 | 0.95 ± 0.05 |
|  | SGF, 24 h | 19.41 ± 1.78 | 0.83 ± 0.10 | 0.97 ± 0.04 |
|  | SIF, 24 h | 19.91 ± 1.80 | 0.82 ± 0.10 | 0.96 ± 0.04 |
| Dandelion | Control group | 27.84 ± 4.27 | 0.89 ± 0.09 | 0.97 ± 0.05 |
|  | SGF, 24 h | 27.98 ± 3.75 | 0.90 ± 0.09 | 0.98 ± 0.05 |
|  | SIF, 24 h | 28.29 ± 3.63 | 0.90 ± 0.08 | 0.98 ± 0.07 |
| Lycopodium | Control group | 30.95 ± 3.33 | 0.82 ± 0.11 | 0.95 ± 0.06 |
|  | SGF, 24 h | 31.45 ± 3.07 | 0.82 ± 0.10 | 0.96 ± 0.04 |
|  | SIF, 24 h | 30.92 ± 3.06 | 0.81 ± 0.11 | 0.95 ± 0.05 |
